# Reducing Length of Stay for Pediatric and Adult Patients in a Student-Run Free Clinic: A Quality Improvement Initiative

**DOI:** 10.7759/cureus.90146

**Published:** 2025-08-15

**Authors:** Silvija Milanovic, Tala Sartawi, Sajan Shroff, Kelly Gahagan, Miranda J Reid, Hannah Rains, Michele N Lossius, Carolyn K Holland

**Affiliations:** 1 College of Medicine, University of Florida College of Medicine, Gainesville, USA; 2 Health Outcome and Biomedical Informatics, University of Florida College of Medicine, Gainesville, USA; 3 Pediatrics, University of Florida College of Medicine, Gainesville, USA; 4 Emergency Medicine, University of Florida College of Medicine, Gainesville, USA

**Keywords:** ambulatory care, quality improvement and patient safety, reduce length of stay, student-run clinic, underserved populations

## Abstract

Introduction: Bartley Temple (BT), a church-based clinic within the University of Florida’s Equal Access Clinic Network, provides care to a predominantly pediatric population as well as adult patients who are uninsured or underinsured. Long patient wait times are known to negatively impact patient satisfaction and clinic efficiency. This study aimed to reduce the median length of stay (LOS) at BT by 25%, from a baseline of 108.8 minutes to 81.6 minutes, through three Plan-Do-Study-Act (PDSA) cycles over six months.

Methods: Three four-week PDSA cycles were implemented following baseline data collection for 12 weeks. PDSA 1 restructured officer roles to improve coordination. PDSA 2 introduced a patient tracker and checklist for the check-in volunteers. PDSA 3 optimized volunteer workflow and check-out processes. LOS data was collected using an Excel-based tracking system (Microsoft® Corp., Redmond, WA, USA). Statistical analysis was performed in R version 4.2.2 (The R Foundation for Statistical Computing, Vienna, Austria).

Results: Although the initial goal of decreasing LOS by 25% was not met, the interventions collectively led to a reduction in median LOS, from 108.8 to 94.3 minutes (13.3%). Individually, pediatric patient LOS decreased by 10.2%, while adult patient LOS decreased by 25.0%.

Conclusion: Creating structured officer roles, patient trackers, checklists, and streamlined check-out processes successfully reduces LOS at a student-run clinic for both pediatric and adult patients.

## Introduction

The Bartley Temple (BT) clinic is part of the University of Florida’s student-run Equal Access Clinic Network (EACN), which encompasses four primary care clinics as well as a variety of specialty clinics. EACN provides essential healthcare to vulnerable populations, including both pediatric and adult patients. The network primarily serves patients who are uninsured or underinsured in Gainesville, Florida, and the surrounding areas. There are four primary care clinic sites, half of which are in traditional clinic settings, while the other half are set up in churches in the evenings, including BT. Additionally, BT is the only site within EACN that has a specific focus on pediatric care, where approximately 64% of our patients are under the age of 18 [1]. While BT plays a crucial role in meeting the healthcare needs of this population, prolonged waiting times have been a persistent issue, negatively impacting patient satisfaction and clinic efficiency [2]. Because this intervention was implemented in a church-based clinic and in a predominantly pediatric population, our findings may have unique implications. These factors may limit generalizability to other settings, but they may also broaden applicability to clinics serving similar populations or operating in nontraditional spaces.

Long wait times and the negative impact on patient satisfaction are not unique to BT or other student-run free clinics (SRFCs). Surveys of patient satisfaction at SRFC predominantly cite waiting time as the metric with the lowest patient satisfaction [3-6]. Prolonged wait times in hospitals and other healthcare facilities have also long been reported as a critical factor that negatively impacts patient satisfaction as well as the perceived quality of care and the operational performance of hospitals [7]. Downstream effects of long wait times include diminished perceptions of information, instructions, and overall treatment received by patients, which may lead to decreased adherence to established care plans or decreased likelihood of returning for follow-up healthcare needs [1-8]. Therefore, identifying bottlenecks and optimizing workflows without compromising quality of care should be a priority across the healthcare system, especially for student-run clinics.

Research has shown that structured workflows, role clarity, and staff coordination can significantly reduce patient wait times and improve operational efficiency [9]. Using this information, a quality improvement project was conducted within a broader multi-phase continuous quality improvement project launched by EACN with the aim of decreasing total patient visit length across all clinic sites. BT set a goal to reduce the median patient length of stay (LOS) by 25% over six months, from a baseline of 108.8 minutes to 81.6 minutes, to address the issue of long wait times. This goal was to be achieved through multiple Plan-Do-Study-Act (PDSA) cycles, using interventions designed to optimize officer workflow and clarify role assignments within the clinic, beginning in the summer of 2022.

Below, we describe the experience and outcome of this project at BT. This manuscript was prepared in accordance with the SQUIRE 2.0 (Standards for Quality Improvement Reporting Excellence) guidelines [10]. This study builds on previous work to address LOS at student-run clinics by evaluating a different set of strategies and testing these strategies in a majority pediatric population [11]. Additionally, given its implementation in a church-based clinic, this study may also offer insights for other clinics operating in nontraditional environments.

This article was presented in poster format at the Institute of Healthcare Improvement (IHI) Forum on December 8-11, 2024.

## Materials and methods

Context

**Figure 1:**
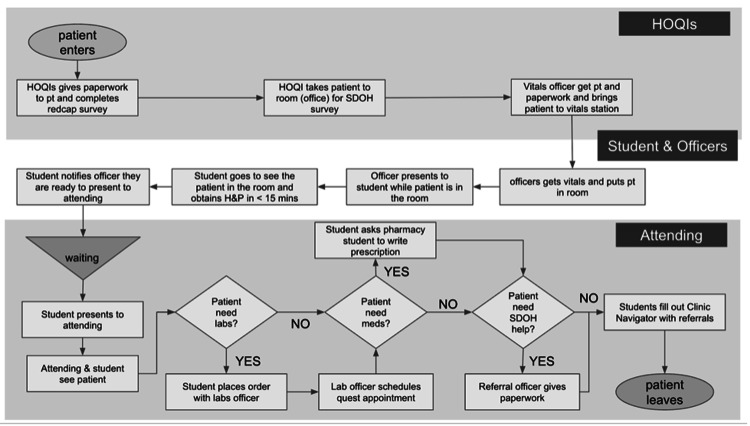
Swimlane Diagram Illustrating Clinic Flow at Bartley Temple Prior to Plan-Do-Study-Act Cycle 1 HOQI: Health Outreach and Quality Improvement; pt: patient; SDOH: social determinants of health; H&P: history and physical Image Credit: Silvija Milanovic and Tala Sartawi

BT clinic runs weekly on Wednesdays with the help of four primary teams: a medical student officer team, an undergraduate Health Outreach and Quality Improvement (HOQI) volunteer team, a pharmacy student team, and two physicians (one adult and one pediatric provider). The clinic is managed by a medical student clinic director and a group of five clinic officers who help with the flow of the clinic, which is described using the Swimlane Diagram (Figure 1).

The clinic flow begins with (1) check-in, managed by HOQI volunteers. After signing in and completing paperwork, the HOQI guides the patient to the vitals station (2). During vitals collection, the HOQI presents the patient to their assigned volunteer (3). After vitals, the patients are escorted to their room (4). The student volunteer then conducts a 15-minute visit (student seeing) (5) and presents the case to the physician (6). There is often a wait period between 5 and 6 (waiting). Together, the provider and student revisit the patient (medical doctor (MD) seeing) (7). Afterward, the student handles labs or prescriptions as needed (8). Once the patient receives all required referrals and prescriptions, the visit is concluded (9). The LOS includes the sum of all of these steps, from the minute the patient checks in to the time they check out.

Interventions

**Figure 2:**
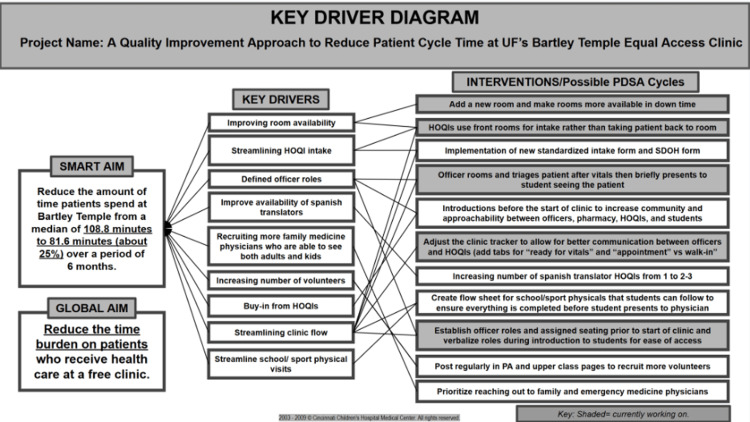
Key Driver Diagram Illustrating the Selected Interventions Implemented During Each Plan-Do-Study-Act (PDSA) Cycle HOQI: Health Outreach and Quality Improvement; SDOH: social determinants of health; PA: physician assistant Image Credit: Silvija Milanovic and Tala Sartawi

To address the issue of prolonged patient LOS, PDSA cycles were utilized to introduce workflow improvements. Prior to starting the PDSA cycles, a key driver analysis was conducted to help identify the main areas that require improvement (Figure 2).

After analysis, a number of issues were selected and addressed by three PDSA cycles, each spanning the duration of four weeks, with LOS chosen as the main outcome measure.

PDSA cycle 1: restructuring officer roles

The first intervention focused on assigning officers specific roles such as check-in, vitals, volunteer coordination, and labs/referrals. Each officer was assigned a dedicated seat and station, moving away from a central officer table. This change was aimed at reducing time spent in unnecessary coordination and streamlining the clinic flow while improving communication and organization.

PDSA cycle 2: HOQI team internal tracker

The second intervention targeted the HOQI team by introducing a structured internal tracker (Excel-based (Microsoft® Corp., Redmond, WA, USA)) that assigned specific patients to individual HOQI volunteers. This tracker allowed volunteers to organize the check-in process into discrete steps and record the time spent at each station, providing clear roles while improving efficiency. The tracker allowed for communication between HOQI volunteers without confusion or need for clarification on who was responsible for each task.

PDSA cycle 3: optimizing volunteer workflow

The third intervention focused on optimizing the patient check-out process and improving volunteer coordination. A “Volunteer Checklist” with time stamps was introduced to guide task completion efficiently. A standardized, concise pre-clinic speech covered key details on clinic flow, time management, and quality improvement goals. To minimize clinic inefficiencies, the labs officer was relocated to sit with the pharmacy student, and a checklist for checkout officers was implemented to reduce volunteer confusion. Arrival times were adjusted to have officers and volunteers show up 30 minutes earlier than before to reduce bottlenecks and improve initial setup. The pre-clinic email was updated to communicate clinic flow adjustments, all contributing to further reductions in patient wait times.

Measures

Patient LOS was selected as the primary metric to assess the impact of the intervention. All patient visits with complete time stamps from check-in through check-out were eligible for inclusion. Visits were excluded if patients left before completing the encounter or if time stamps were incomplete. LOS data were recorded using the pre-existing Excel-based tracking system used across all EACN clinics. This tracker has been in use for multiple years and is maintained by the EACN leadership. Data entry was performed in real time by trained HOQI volunteers and medical student officers, and checked by an on-duty quality improvement officer. Spot checks for accuracy were conducted by the quality improvement team on a weekly basis.

LOS were established for the overall clinic population, as well as separately for adult and pediatric patients. Baseline LOS was determined using a 12-week median from 67 patients before any interventions were implemented. For each PDSA cycle, LOS data, including time at each checkpoint, were tracked for four weeks using a pre-existing Excel system used across all EACN clinics. Weeks were included if LOS data were collected on at least 80% of patients present that night. The number of patients, new patients, translator use, and patient visit type were also collected. Due to a non-normal distribution of LOS, the median and interquartile range (IQR) were calculated for each time interval for each PDSA cycle using R version 4.2.2 (The R Foundation for Statistical Computing, Vienna, Austria).

Analysis

**Figure 3:**
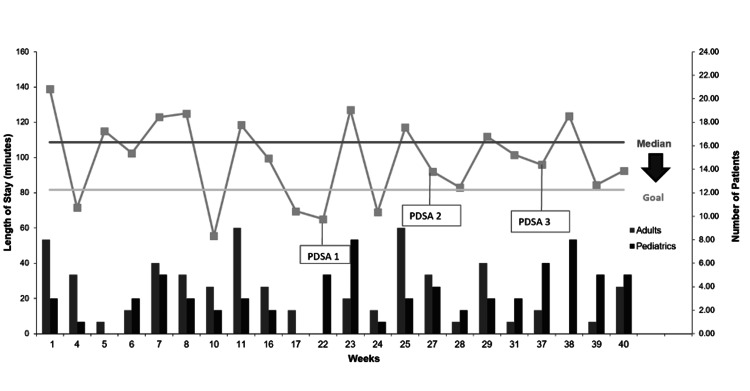
Run Chart Demonstrating Overall Clinic Length of Stay and Patient Volume Over Time With Plan-Do-Study-Act (PDSA) Interventions Image Credit: Tala Sartawi

Descriptive and inferential statistical methods were used to evaluate the impact of PDSA cycle interventions on clinic operations. Process measures, including the number of patients, new patients, and patient visit types, were analyzed using medians and IQRs. Outcome measures focused on visit length, with Pearson correlation coefficients (r) used to examine associations between weekly median visit length and factors such as total patient volume, new patients, patients using translators, pediatric patients, and patients presenting for physicals. To visualize trends over time, a run chart was used to compare baseline median LOS data with post-intervention results (Figure 3), allowing for real-time assessment of system changes and guiding iterative improvements.

Ethical consideration

This project is IRB exempt as a quality improvement project and was thus registered with the UF Health Sebastian Ferrero Office of Clinical Quality and Patient Safety under project identification number 2295.

## Results

The interventions implemented during each PDSA cycle improved patient median LOS and clinic flow. The specifics for each PDSA cycle are highlighted in Table 1.

**Table 1:** Patient Volume and Length of Stay Reduction by Plan-Do-Study-Act (PDSA) Cycle

PDSA	Total Patients	Pediatric Patients, n (%)	Adult Patients, n (%)	Overall (min, %)	Pediatrics (min, %)	Adults (min, %)
Baseline	68	23 (34)	45 (66)	108.8 (-)	105.0 (-)	112.0 (-)
PDSA 1	31	17 (55)	14 (45)	93.0 (14.5)	88.5 (15.7)	111.0 (0.9)
PDSA 2	25	12 (48)	13 (52)	96.8 (11.0)	100.5 (4.2)	87.5 (21.9)
PDSA 3	31	24 (77)	7 (23)	94.3 (13.3)	94.3 (10.2)	84.0 (25.0)

A total of 155 patients were seen in the 12-week period during which the PDSAs were implemented. Among the 155 patients seen during the interventions, 76 (49%) were pediatric patients, with 49 (64%) of the pediatric visits focusing on school and sport physicals (29 (38%), 20 (26%), respectively). After PDSA 1-3, the overall clinic median LOS decreased by 13.3%, from 108.8 to 94.3 minutes. Pediatric LOS decreased by 10.2%, from 105.0 to 94.3 minutes, while adult LOS saw a greater reduction of 25.0%, from 112.0 to 84.0 minutes.

During PDSA 1, which focused on restructuring officer roles, 31 patients were seen over the four weeks, 17 (55%) of whom were pediatric patients and 14 (45%) were adults. The intervention led to a notable 14.5% reduction in median LOS of all patients, decreasing from a baseline of 108.8 to 93.0 minutes. There was a 15.7% reduction in pediatric LOS, while adults only saw a 0.9% decrease. The workflow adjustments, such as assigning officers clearly defined roles and minimizing coordination delays, were key contributors to this initial reduction in LOS.

During PDSA 2, 25 patients were seen over the four weeks, with 12 (48%) pediatric and 13 (52%) adults. The implementation of the HOQI team’s internal tracker resulted in a 11.0% reduction in median LOS from the baseline. There was a 4.2% reduction in pediatric LOS as compared to a 21.9% reduction in adult LOS. These results highlight that streamlining the check-in process was especially effective in enhancing clinic efficiency.

During PDSA 3, which focused on optimizing clinic flow further by improving checkout processes and supporting volunteer coordination, 31 patients were seen over the four weeks, 24 (77%) of whom were pediatric patients and seven (23%) were adults. The intervention resulted in a 13.3% reduction in the overall median LOS from baseline. Pediatric LOS improved by 10.2%, while adult LOS saw a significant 25% reduction. The implementation of a standardized check-out protocol and improved communication between volunteers and officers played a crucial role in these efficiency gains.

Table 2 shows the median number of patients and physicians present per night during each PDSA cycle, as well as the statistical analysis of variations in these characteristics.

**Table 2:** Patient and Provider Characteristics per Night by Plan-Do-Study-Act (PDSA) Cycle (Median (Interquartile Range))

PDSA Cycle	0	1	2	3	χ^2^	df	p-value
Patients	6.00 (5.25, 10.25)	8.00 (4.50, 11.25)	6.50 (3.75, 9.00)	8.00 (7.50, 8.25)	0.53442	3	0.9113
New patients	2.50 (1.25, 3.75)	4.00 (3.00, 4.25)	3.00 (2.75, 4.00)	5.00 (5.00, 6.00)	5.0834	3	0.1658
Pediatric patients	2.50 (1.25, 3.00)	4.00 (2.50, 5.75)	3.00 (2.75, 3.25)	5.50 (5.00, 6.50)	9.186	3	0.02692
School physicals	1.00 (0.00, 1.75)	1.50 (0.75, 2.50)	0.50 (0.00, 1.25)	2.00 (1.00, 3.00)	2.7869	3	0.4257
Sports physicals	1.00 (0.00, 1.75)	0.00 (0.00, 0.50)	0.00 (0.00, 0.25)	2.00 (0.75, 3.25)	4.0878	3	0.2521
Patients using translation	1.50 (1.00, 2.75)	4.00 (1.75, 6.25)	3.50 (2.75, 4.50)	3.00 (2.50, 3.50)	3.3813	3	0.3365
Providers	2.00 (2.00, 2.00)	2.00 (2.00, 3.00)	2.00 (2.00, 2.25)	2.00 (2.00, 2.00)	2.7918	3	0.4249

The number of pediatric patients per night was the only characteristic with statistically significant variation across cycles (p <0.05). The median number of pediatric patients increased from 2.50 during the baseline data collection period to 5.50 in PDSA 3. However, no significant differences were found in the total number of patients, new patients, school or sports physicals, or patients using translation services across cycles.

Table 3 summarizes the median time for each step in the clinic flow per patient per night during each PDSA cycle.

**Table 3:** Median Time per Clinic Step by Plan-Do-Study-Act (PDSA) Cycle (Minutes (Interquartile Range)) MD: medical doctor

PDSA Cycle	0	1	2	3	χ2	df	p-value
Intake	8.00 (5.00, 10.00)	7.50 (6.75, 8.00)	11.75 (10.75, 68,62)	5.75 (3.38, 8.75)	5.8377	3	0.1198
Student seeing	16.00 (10.50, 20.00)	18.50 (12.38, 23.75)	23.00 (19.50, 23.25)	24.50 (18.12, 29.12)	2.6746	3	0.4446
Waiting	15.50 (12.50, 20.50)	16.50 (11.75, 23.62)	19.50 (14.12, 23.62)	21.00 (17.50, 30.00)	1.1185	3	0.7726
MD seeing	22.00 (20.50,25.00)	23.75 (19.50, 28.62)	20.00 (16.88, 24.75)	16.00 (14.88, 18.38)	2.7536	3	0.4312
Total time	108.75 (78.50, 121.88)	93.00 (68.00, 119.50)	96.75 (89.75, 104.12)	94.25 (90.50, 102.88)	0.60119	3	0.8962

No statistically significant variation in time intervals between cycles was found for intake, student seeing, waiting, or MD seeing steps (all p > 0.05). Despite the lack of statistical significance, the total time per patient decreased from a baseline of 108.75 minutes to 94.25 minutes by PDSA 3, indicating a general trend toward improved efficiency across cycles.

Correlational analysis revealed several relationships between clinic characteristics and the median weekly visit length. Weak positive relationships were found between the median LOS and the number of sports physicals (r = 0.24) and the number of patients using translation services (r = 0.37), indicating a modest increase in LOS with more of these visits. A moderate positive relationship was noted between the number of school physicals (r = 0.44) and pediatric patients (r = 0.53) and the median LOS, with both factors contributing to longer visits. Lastly, a strong positive relationship was observed between the total number of patients and the median LOS (r = 0.60), indicating that increased patient volume significantly impacted visit length.

## Discussion

Interpretation

The outcomes of the PDSA cycles demonstrate the importance of structured workflows, communication tools, and consistent staffing in optimizing clinic efficiency. Clearly defined roles for clinic officers helped improve coordination and reduce delays, reinforcing findings from prior literature. A study conducted in a urology procedures clinic found that assigning triage and procedural roles led to a 42% reduction in wait times, underscoring the value of parallel task execution and responsibility delineation [12]. Although that study occurred in a specialty clinic setting, the principle of enhancing efficiency through simultaneous workflows is applicable across diverse outpatient environments, including student-run clinics. In our setting, role clarity likely helped mitigate delays caused by inexperience or uncertainty among volunteers, particularly during high-volume periods. However, the potential influence of the Hawthorne effect cannot be excluded. In our setting, student volunteers were aware of the quality improvement project overall and PDSA 3 as it directly involved their participation, but were not aware of the interventions of PDSA 1 and 2. Faculty were aware of the quality improvement project but were not informed of specific interventions, as their role was not altered. The Hawthorne effect was therefore unavoidable, and observers were not blinded to intervention status. To minimize bias, we maintained consistent data collection procedures throughout the study period and ensured the same officer roles were responsible for recording LOS across PDSA cycles.

Effective communication tools were another key contributor to improved flow. The introduction of the internal HOQI tracker provided real-time visibility of patient progress and responsibilities, which proved especially helpful for newer volunteers and patients requiring more extensive intake procedures. This aligns with previous studies demonstrating that electronic patient tracking tools enhance team coordination and streamline task distribution [13,14]. However, our current electronic medical record (EMR) system lacks many of the features available in more sophisticated platforms. Greater integration of tracking functionalities into the EMR may offer further gains, particularly as the clinic continues to serve a high volume of walk-in patients with variable needs.

Interventions targeting student volunteers also contributed meaningfully to reductions in LOS. However, the weekly turnover of both volunteers and attending physicians introduced variability in familiarity with clinic workflows and in the consistent application of new processes. Differences in clinical experience, comfort with the setting, and adherence to updated protocols likely contributed to the degree of variability observed in LOS. These findings mirror prior literature identifying inconsistent staffing as a barrier to sustaining quality improvement initiatives in SRFCs [15,16]. While our analysis did not show a statistical association between provider number and visit duration, factors such as provider specialty and familiarity with BT may still have influenced clinic flow and should be explored in future studies.

Volunteer turnover is a well-recognized barrier to sustaining improvements in SRFC settings. Rupert et al. describe annual student leadership turnover and frequent changes in volunteer physicians as self-reported weaknesses that can delay or disrupt ongoing initiatives [16]. In contrast, BT maintained consistent officer roles responsible for data tracking and workflow oversight across all PDSA cycles. This structure likely mitigated the impact of rotating student and faculty volunteers, enabling greater continuity in implementing and reinforcing interventions compared with other SRFCs facing similar turnover challenges.

Additionally, external factors such as patient demographics and visit complexity may have influenced outcomes. The rise in pediatric visits throughout the study, without an accompanying increase in provider number, likely contributed to a variable patient-to-provider ratio and longer visits. Pediatric patients often appeared to require more time for physicals and common point-of-care testing such as hemoglobin and urine dipstick checks. However, specific timing data for these portions of the visit were not collected, and future studies should examine section-level visit times to better quantify these differences. Similarly, adult patients with more complex concerns or lab needs also seemed to experience longer visits. These observations are consistent with findings from a student-run clinic at Ohio State University, which reported that visit length increased significantly for patients with higher medical complexity or those requiring labs and referrals [17]. These factors highlight the importance of considering patient mix and service intensity when evaluating efficiency interventions.

Pediatric LOS improved by 10.2%, while adult LOS saw a significant 25% reduction. Several factors may explain this difference. Pediatric visits at BT often required additional components such as school or sports physicals, growth charting, and point-of-care testing (e.g., hemoglobin, urine dipstick) as noted above, which extended visit duration and were not individually targeted by our interventions. Pediatric encounters also tended to involve more time spent communicating with both patients and parents or guardians. In contrast, adult visits during the study period were often less procedurally intensive and could be addressed more efficiently within the streamlined workflow. The increase in pediatric patient volume without a corresponding rise in provider numbers may have further amplified these effects by disproportionately impacting the patient-to-provider ratio for pediatric care.

Limitations

Clinic operations were influenced by several structural limitations. Visit duration varied with volunteer experience, as shifts included first- through fourth-year medical students. Less experienced students generally required more time, affecting efficiency, consistent with prior findings that experience impacts care quality and flow [11]. Officer absences due to exams or clinical rotations disrupted key tasks such as data tracking and training, especially during PDSA 1, which relied heavily on officer-led processes. Staffing inconsistency is a known barrier to sustaining improvements [15,16]. Physician familiarity with BT also mattered; attendings with prior experience at BT, other EACN clinics, or in outpatient settings adapted quickly, while new attendings required real-time orientation, which slowed operations. Although provider count remained stable and was not statistically linked to visit duration, unmeasured factors like specialty and SRFC familiarity likely influenced flow. Later PDSA cycles saw a rise in pediatric patients (Table 1) without a change in provider number (Table 2), leading to fluctuating patient-to-provider ratios and longer visits. The walk-in model limited predictability, highlighting the need to consider volume and distribution when assessing efficiency. While LOS was measured as the primary outcome, patient satisfaction - a key downstream effect of reduced wait times - was not assessed before or after the interventions. Future work should incorporate patient satisfaction measures to better understand the broader impact of workflow changes. Finally, patient complexity impacted LOS, as shown in an Ohio State student-run clinic study linking medical complexity and use of services like point-of-care testing (POCT), labs, or referrals to longer visits [17]. At BT, adult patients were often more complex, while pediatric visits commonly included POCT (e.g., hemoglobin checks, urine dipsticks), further extending time [17].

The sustainability of these improvements beyond the study period was not formally assessed; however, monitoring of LOS has continued following project completion, and the workflow changes implemented remain in place given their observed benefits. BT now has a designated quality improvement officer and team, which will continue to track LOS and develop additional quality improvement initiatives aimed at further improving both LOS and patient satisfaction.

## Conclusions

Although the target LOS reduction was not fully met, the interventions led to meaningful improvements in clinic efficiency. These improvements highlight the value of defined roles, structured workflows, better task coordination, and clear expectations for student volunteers. Nonetheless, external variables, including provider specialty, patient complexity, and population distribution, played significant roles in determining patient flow and LOS. These factors should be integrated into the planning of future interventions to further enhance clinic performance. With strong positive feedback from patients, volunteers, and staff, the clinic is well-positioned to continue refining its operations. Going forward, improving consistency in staffing and adapting processes to better manage pediatric patients will be key steps in achieving sustained LOS reductions.
